# Supplementary material for: Multimorbidity Patterns and Unplanned Hospitalisation in a Cohort of Older Adults
Source: J Clin Med. 2020 Dec 10;9(12):4001. doi: 10.3390/jcm9124001 (PMC7764652; doi:10.3390/jcm9124001)
Supplement: Supplementary file 1 [file jcm-09-04001-s001.pdf]

Table S1. Baseline prevalence (%) of chronic diseases in the study population.

**Chronic disease**	**%**	**Chronic disease**	**%**
Hypertension	81.89	Neurotic, stress-related and somatoform diseases	3.43
Dyslipidaemia	55.49	Sleep disorders	2.54
Chronic kidney diseases	37.07	Migraine and facial pain syndromes	2.45
Obesity	14.54	Other genitourinary diseases	2.27
Osteoarthritis and other degenerative joint diseases	13.87	Other cardiovascular diseases	2.23
Ischemic heart disease	12.85	Cardiac valve diseases	2.01
Thyroid diseases	11.33	Allergy	1.96
Deafness, hearing impairment	11.28	Other neurological diseases	1.92
Anemia	10.53	Chronic pancreas, biliary tract and gallbladder diseases	1.47
Depression and mood diseases	9.63	Other metabolic diseases	1.47
Diabetes	9.28	Other psychiatric and behavioural diseases	1.29
Atrial fibrillation	8.25	Peripheral vascular disease	1.16
Heart failure	8.07	Bradycardias and conduction diseases	1.12
Solid neoplasms	8.03	Ear, nose, throat diseases	1.03
Dorsopathies	6.96	Other respiratory diseases	0.98
Osteoporosis	6.87	Inflammatory bowel diseases	0.98
Asthma	6.82	Parkinson and parkinsonism	0.94
Cataract and other lens diseases	6.24	Peripheral neuropathy	0.94
Other musculoskeletal and joint diseases	5.89	Venous and lymphatic diseases	0.80
Glaucoma	5.89	Haematological neoplasms	0.58
Cerebrovascular disease	5.71	Chronic ulcer of the skin	0.58
Other eye diseases	5.40	Other digestive diseases	0.58
Dementia	5.04	Epilepsy	0.54
Colitis and related diseases	4.77	Schizophrenia and delusional diseases	0.54
COPD, emphysema, chronic bronchitis	4.64	Blood and blood forming organ diseases	0.45
Prostate diseases	4.55	Chronic infectious diseases	0.45
Autoimmune diseases	4.37	Other skin diseases	0.18
Oesophagus, stomach, and duodenum diseases	4.06	Chronic liver diseases	0.18
Inflammatory arthropathies	3.75	Multiple sclerosis	0.09
Blindness, visual impairment	3.70	Chromosomal abnormalities	0.00

Abbreviations: COPD – chronic obstructive pulmonary disease.

Table S2. Multimorbidity patterns identified through fuzzy c-mean cluster analysis.

Multimorbidity pattern	Diseases	Prevalence within pattern (%)	Observed/ Expected Ratio	Exclusivity (%)
**Psychiatric**				
	Neurotic, stress-related and somatoform diseases	54.45	15.55	94.52
	Depression and mood diseases	82.57	8.20	49.86
	Other neurological diseases	6.00	2.89	17.55
	Asthma	17.54	2.45	14.91
	Sleep disorders	5.48	2.28	13.84
	Colitis and related diseases	26.82	2.23	13.59
	Migraine and facial pain syndromes	4.95	1.97	11.95
	Other musculoskeletal and joint diseases	12.50	1.80	10.97
	COPD, emphysema, chronic bronchitis	9.12	1.65	10.06
	Oesophagus, stomach and duodenum diseases	7.86	1.58	9.63
**Cardiovascular, Anemia & Dementia**				
	Bradycardias and conduction diseases	18.88	9.08	79.84
	Other cardiovascular diseases	25.51	6.92	60.87
	Heart failure	71.59	6.52	57.32
	Cardiac valve diseases	17.82	6.11	53.68
	Atrial fibrillation	58.23	5.54	48.73
	Cerebrovascular disease	26.74	3.39	29.84
	Ischemic heart disease	51.85	3.07	26.99
	Anemia	37.25	2.84	25.00
	Dementia	17.20	2.84	24.97
	Inflammatory arthropathies	11.71	2.57	22.57
**Sensory & Cancer**				
	Cataract and other lens diseases	35.98	5.87	68.86
	Blindness, visual impairment	24.40	5.72	67.06
	Other eye diseases	33.09	5.70	66.92
	Glaucoma	26.79	4.42	51.89
	Deafness, hearing impairment	34.82	3.00	35.21
	Solid neoplasms	21.93	2.15	25.19
	Other genitourinary diseases	5.67	1.97	23.08
	Dementia	11.83	1.95	22.91
	Anemia	22.77	1.74	20.39
	Chronic kidney diseases	65.36	1.69	19.85
**Metabolic & Sleep**				
	Diabetes	37.96	3.77	42.31
	Sleep disorders	7.62	3.16	35.50
	Obesity	38.64	2.79	31.28
	Ischemic heart disease	33.50	1.98	22.26
	Inflammatory arthropathies	9.02	1.98	22.20
	Other cardiovascular diseases	6.34	1.72	19.31
	Dementia	10.29	1.70	19.08
	Prostate diseases	7.56	1.63	18.30
	COPD, emphysema, chronic bronchitis	8.17	1.48	16.65
	Heart failure	16.13	1.47	16.49
**Musculoskeletal, Respiratory & Gastro-intestinal**				
	Osteoporosis	31.41	4.26	67.29
	Dorsopathies	21.80	2.93	46.23
	Oesophagus, stomach and duodenum diseases	14.21	2.86	45.20
	Asthma	19.98	2.79	44.10
	COPD, emphysema, chronic bronchitis	12.77	2.32	36.59
	Autoimmune diseases	11.67	2.28	36.07
	Inflammatory arthropathies	9.07	1.99	31.39
	Thyroid diseases	22.74	1.96	30.95
	Colitis and related diseases	22.95	1.91	30.19

	Osteoarthritis and other degenerative joint diseases	27.58	1.88	29.61
**Unspecific**				
	Dyslipidemia	67.35	1.28	59.30
	Hypertension	84.61	1.12	51.82
	Obesity	13.07	0.94	43.71
	Prostate diseases	4.29	0.93	42.92
	Other musculoskeletal and joint diseases	5.83	0.84	39.00
	Other genitourinary diseases	2.42	0.84	38.96
	Solid neoplasms	7.86	0.77	35.69
	Migraine and facial pain syndromes	1.88	0.75	34.64
	Thyroid diseases	8.56	0.74	34.23
	Osteoarthritis and other degenerative joint diseases	10.63	0.72	33.53

Abbreviations: COPD – chronic obstructive pulmonary disease.

Table S3. Distribution of deaths during follow-up by multimorbidity pattern.

	Total population N=2,250	Psychiatric n=132 (5.9%)	Cardio/Anemia/Dementia n=141 (6.3%)	Metabolic/Sleep n=240 (10.7%)	Sensory/Cancer n=267 (11.9%)	MSK/Resp/GI n=355 (15.7%)	Unspecific n=1,115 (49.5%)
**Alive after 5 years (%)**	1,856 (82.5)	111(84.1)	68 (48.2)	200 (83.3)	173 (64.8)	290 (81.7)	1,014 (90.9)
**Died during 1st year (%)**	58 (2.6)	3 (2.3)	18 (12.8)	2 (0.8)	11 (4.1)	8 (2.3)	16 (1.4)
**Died during 2nd year (%)**	81 (3.6)	3 (2.3)	14 (9.9)	14 (5.8)	17 (6.4)	13 (3.7)	20 (1.8)
**Died during 3rd year (%)**	81 (3.6)	4 (3.0)	14 (9.9)	6 (2.5)	22 (8.2)	13 (3.7)	22 (2.0)
**Died during 4th year (%)**	83 (3.7)	4 (3.0)	16 (11.4)	7 (2.9)	20 (7.5)	13 (3.7)	23 (2.1)
**Died during 5th year (%)**	91 (4.0)	7 (5.3)	11 (7.8)	11 (4.6)	24 (9.0)	18 (5.1)	20 (1.8)

Abbreviations: GI – gastro-intestinal diseases; Resp – respiratory diseases; MSK – musculoskeletal diseases; Cardio – cardiovascular diseases.

Table S4. Association of multimorbidity patterns with unplanned hospital care utilisation stratified by age.

	<78 years		≥78 years	
**Multimorbidity patterns**	**IR per 10 person years**	**HR (95% CI)**	**IR per 10 person years**	**HR (95% CI)**
**First hospitalisation**				
Unspecific	0.52	Ref	1.45	Ref
Psychiatric	0.97	1.92 (1.30, 2.84) ***	1.75	1.19 (0.80, 1.77)
Cardio/Anemia/Dementia	1.05	1.86 (0.87, 4.00)	4.03	2.06 (1.58, 2.68) ***
Metabolic/Sleep	0.89	1.55 (1.11, 2.16) **	2.10	1.51 (1.13, 2.01) **
Sensory/Cancer	0.86	1.22 (0.70, 2.13)	2.35	1.20 (0.96, 1.52)
MSK/Resp/GI	0.50	0.95 (0.66, 1.34)	2.09	1.40 (1.01, 1.77) **
**Multimorbidity patterns**	**IR per 10 person years**	**IRR (95% CI)**	**IR per 10 person years**	**IRR (95% CI)**
**Unplanned hospitalisations**				
Unspecific	0.78	Ref	6.08	Ref
Psychiatric	1.85	2.36 (1.43, 3.88) ***	6.14	1.01 (0.73,1.39)
Cardio/Anemia/Dementia	1.97	2.51 (0.95, 6.62)	9.51	1.57 (1.28, 1.92) ***
Metabolic/Sleep	1.75	2.24 (1.50, 3.35) ***	8.19	1.35 (1.08, 1.69) **
Sensory/Cancer	1.35	1.73 (0.88, 3.39)	6.52	1.07 (0.89, 1.28)
MSK/Resp/GI	1.00	1.28 (0.87, 1.88)	5.62	0.92 (0.76, 1.12)
**Days of in-hospital stay**				
Unspecific	5.76	Ref	65.70	Ref
Psychiatric	22.80	3.95 (1.67, 9.33) **	175.65	2.67 (1.29, 5.54) **
Cardio/Anemia/Dementia	11.56	2.00 (0.35,11.50)	126.57	1.93 (1.17, 3.18) *
Metabolic/Sleep	15.02	2.60 (1.26, 5.37) **	77.05	1.17 (0.70, 1.96)
Sensory/Cancer	12.44	2.16 (0.66, 7.07)	59.52	0.91 (0.59, 1.38)
MSK/Resp/GI	7.35	1.27 (0.67, 2.41)	67.60	1.03 (0.68, 1.56)
**30-day readmissions**				
Unspecific	0.08	Ref	0.50	Ref
Psychiatric	0.34	4.08 (1.13, 14.80) *	1.11	2.22 (0.83, 5.92)
Cardio/Anemia/Dementia	0.00	0.00 (0.00, 0.00)	1.54	3.05 (1.61, 5.78) ***
Metabolic/Sleep	0.58	7.02 (2.52, 19.56) ***	1.25	2.48 (1.29, 4.76) **
Sensory/Cancer	0.22	2.63 (0.50, 13.87)	0.67	1.33 (0.76, 2.33)
MSK/Resp/GI	0.18	2.21 (0.82, 5.96)	0.62	1.23 (0.69, 2.19)

P-values: * = < 0.05, ** = < 0.01, ***= < 0.001. Models adjusted by age, sex, education level, alcohol consumption, and smoking. Abbreviations: GI – gastro-intestinal diseases; Resp – respiratory diseases; MSK – musculoskeletal diseases; Cardio – cardiovascular diseases; HR – hazard ratio; IRR – incidence rate ratio; CI – confidence interval; IR – incidence rate.
